# Supplementary material for: Human lung-resident mucosal-associated invariant T cells are abundant, express antimicrobial proteins, and are cytokine responsive
Source: Commun Biol. 2022 Sep 9;5:942. doi: 10.1038/s42003-022-03823-w (PMC9463188; doi:10.1038/s42003-022-03823-w)
Supplement: Supplementary file 1 — Supplementary Information [file 42003_2022_3823_MOESM1_ESM.pdf]

Human Lung-resident Mucosal-Associated Invariant T cells are Abundant, Express Antimicrobial Proteins, and are Cytokine Responsive

Erin W. Meermeier[1], Christina L. Zheng[2], Jessica G Tran[3], Shogo Soma[1], Aneta H. Worley[3], David I. Weiss[4], Robert L. Modlin[5], Gwendolyn Swarbrick[1,3], Elham Karamooz[1,3], Sharon Khuzwayo[6,7], Emily B. Wong[6,7,8,9], Marielle C. Gold[1,3], David M. Lewinsohn[1,3]

[1] Department of Pulmonary and Critical Care Medicine, Oregon Health & Science University, Portland, OR 97239, USA

[2] Department of Medical Informatics and Clinical Epidemiology, Oregon Health & Science University, Portland, OR 97239, USA

[3] VA Portland Health Care System, Portland, OR 97239, USA

[4] David Geffen School of Medicine at UCLA, Los Angeles, CA 90095, USA

[5] Division of Dermatology, Department of Medicine, David Geffen School of Medicine at University of California, Los Angeles, Los Angeles, CA 90095, USA

[6] Africa Health Research Institute, Durban, South Africa

[7] School of Laboratory Medicine and Medical Sciences, University of KwaZulu-Natal, Durban, South Africa

[8] Division of Infectious Diseases, Massachusetts General Hospital, Boston, MA, USA

[9] Harvard Medical School, Boston, MA, USA

[10] Division of Infection and Immunity, University College London, London, UK

Supplemental files:

Supplemental Table 1

Supplemental Figure 1-5

Donor number	Cause of Death	Gender	Age	BMI (kg/m^2)
OD98	Cardiovascular anoxia	F	60	40.8
OD101	Cerebrovascular/Stroke	F	64	27.5
OD103	Head Trauma	M	12	14.4
OD104	Anoxia	F	15	29.7
OD105	Cerebrovascular/Stroke	M	11	13.7
OD107	Cerebrovascular/Stroke	M	67	25.3
OD111	Anoxia	F	22	22.5
OD112	Cerebrovascular/Stroke	F	60	33.5
OD113	Cerebrovascular/Stroke	M	69	22.4
OD114	Cerebrovascular/Stroke	M	56	17.6
OD116	Anoxia	M	10	17.3
OD117	Cerebrovascular/Stroke	F	67	32.1
OD119	Cerebrovascular/Stroke	F	58	26.4
OD120	Anoxia	F	39	16.9
OD121	Anoxia	F	6	20.7
OD123	Cerebrovascular/Stroke	M	39	29.3
OD126	Cerebrovascular/Stroke	M	22	18.6
OD128	Cerebrovascular/Stroke	F	62	28.2
OD133	Anoxia	M	31	31
OD144	Cardiovascular anoxia	M	51	32.5

Supplemental Table 1. Pacific Northwest Transplant Bank donors provide lung tissue for single T cell analyses. Donor lung tissue was accepted for our study based on criteria detailed in the methods section. OD103, 104, 105, and 107 were used for RNA-seq analysis. The remaining lung donors were used for validation experiments.

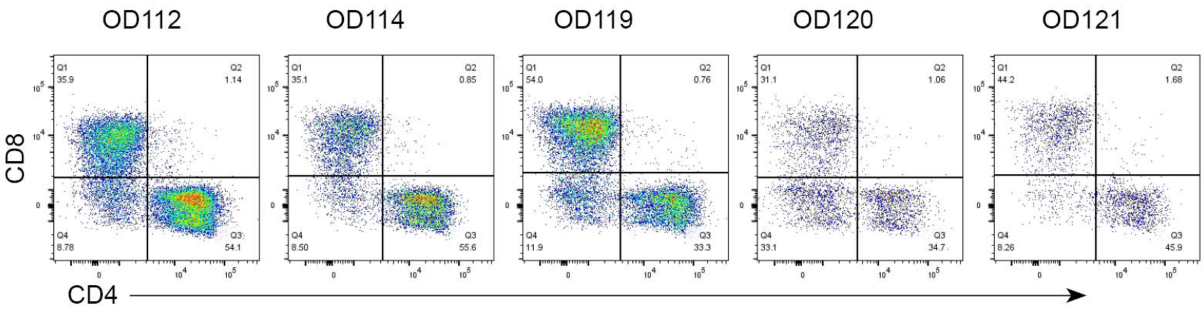

Supplemental Figure 1. Lung MAIT cells express CD8 and CD4. Flow cytometry on lung samples to measure CD4 and CD8 co-receptor expression. Lung cells were rested overnight and expression on all $CD3^{+}$ T cells are shown.

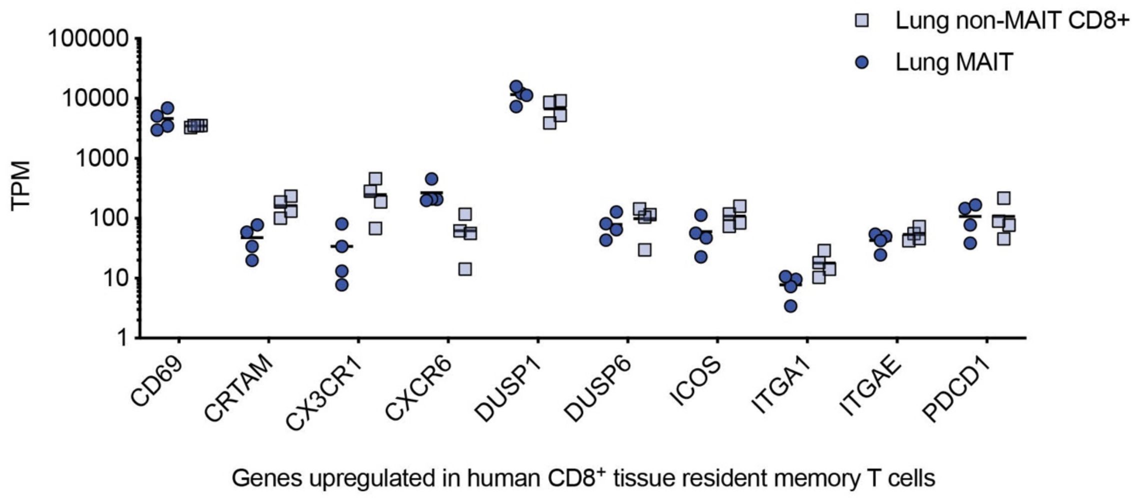

Supplemental Figure 2. Lung MAIT cells express genes associated with human tissue-resident memory CD8⁺ T cells. Transcripts per million (TPM) reads from MR1/5-OP-RU⁺ CD8⁺ T cells derived from lung samples (Lung MAIT, blue filled circles), and MR1/5-OP-RU negative CD8⁺ T cells derived from lung samples (Lung non-MAIT CD8⁺, blue open squares). The mean of each group of samples is shown as a black bar. Genes listed on the x-axis are grouped by whether they have been observed as upregulated (A) in human CD8⁺ tissue-resident memory T cells compared to non-tissue resident memory T cells.

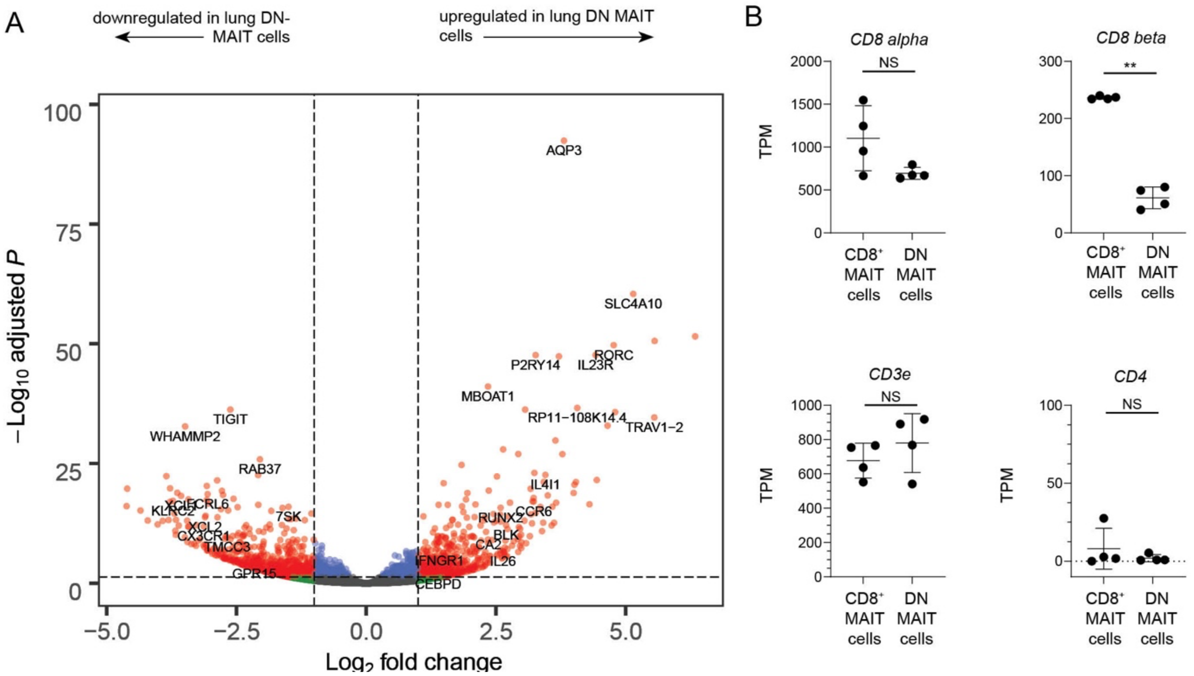

Supplemental Figure 3. Differentially expressed genes of CD8$^-$CD4$^-$ MAIT cells in the lung.

A. A volcano plot displays the differentially expressed genes between lung derived CD8-CD4- MAIT cells (on the right) and lung-derived CD8-CD4- lymphocytes (left) . Significantly differentially expressed genes (log2 fold change >1 or <-1, and FDR p-value ≤ 0.05) are represented as red. B. Box plots of transcript per million (TPM) reads of genes commonly associated with MAIT cells, from RNA-seq samples of CD8+ MAIT cells or non-MAIT CD8+ T cells derived from four lung samples. Box plots indicate the median and range of the TPM RNA-seq reads. FDR *P < 0.05, **P < 0.01, ****P < 0.0001.

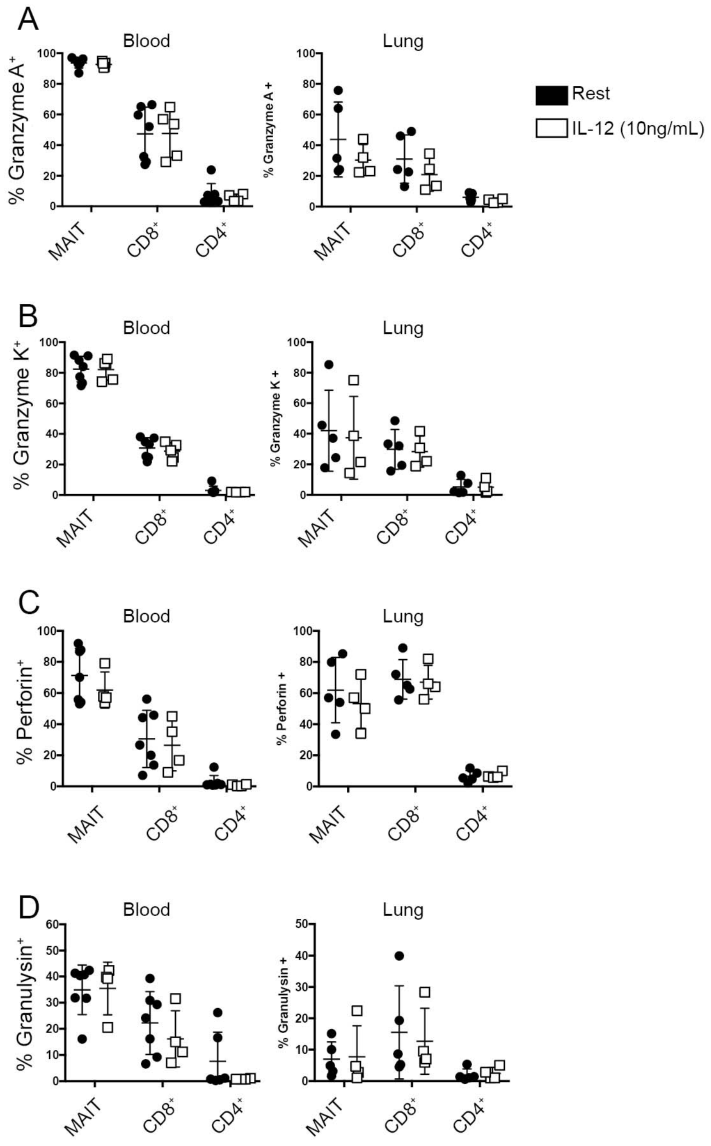

Supplemental Figure 4. Poised expression of cytolytic proteins by subset of human T cells in the lung compared to the blood. Cells derived from PBMC or lung samples were either rested (black squares) overnight or in the presence of IL-12 cytokine (white squares, 10 ng/mL) and then stained intracellularly for (A) granzyme A, (B) granzyme K, (C) perforin, (D) granulysin. The frequency of cells staining positively for each protein is plotted from 5 PBMC donors (left) and 5 lung donors (right). Representative results of three experiments are shown, n = 5 biological replicates.

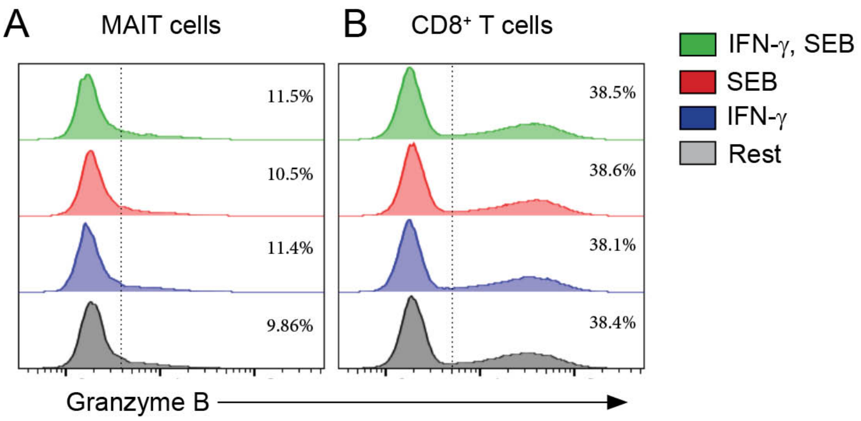

Supplemental Figure 5. Intracellular levels of granzyme B in MAIT cells in the context of TCR stimulation or IFN-γ. Cells derived from PBMC were either rested (gray histograms) overnight or in the presence of IFN-γ (blue histograms, 10 ng/mL), SEB (red histograms), or IFN-γ and *Staphylococcus* enterotoxin B (SEB) (green histograms) and then stained intracellularly for granzyme B. The frequency of granzyme B^+ MR1-tetramer$^+$ MAIT cells (A) or non-MAIT $CD8^+$ T cells (B) from one of five representative donors is plotted. Representative results of two experiments are shown.
