# Supplementary material for: Human lung-resident mucosal-associated invariant T cells are abundant, express antimicrobial proteins, and are cytokine responsive
Source: Commun Biol. 2022 Sep 9;5:942. doi: 10.1038/s42003-022-03823-w (PMC9463188; doi:10.1038/s42003-022-03823-w)
Supplement: Supplementary file 2 — Description of Additional Supplementary Files [file 42003_2022_3823_MOESM2_ESM.pdf]

Description of Additional Supplementary Files

File name: Supplemental Data 1
Description: The source data behind the graphs in the paper

File name: Supplemental Data 2
Description: Differential gene expression of lung derived MAIT cells and non-MAIT CD8+ T cells.

Genes that were enriched in MAIT samples from lung (compared to lung CD8+ non-MAIT) at thresholds of FDR-adjusted P value ≤ 0.001; and log2 fold change > 1 or <-1.
