# Supplementary material for: Human lung-resident mucosal-associated invariant T cells are abundant, express antimicrobial proteins, and are cytokine responsive
Source: Commun Biol. 2022 Sep 9;5:942. doi: 10.1038/s42003-022-03823-w (PMC9463188; doi:10.1038/s42003-022-03823-w)
Supplement: Supplementary file 5 — Reporting summary [file 42003_2022_3823_MOESM5_ESM.pdf]

nature research

Corresponding author(s): David Lewinsohn

Last updated by author(s): Jun 29, 2022

Reporting Summary

Nature Research wishes to improve the reproducibility of the work that we publish. This form provides structure for consistency and transparency in reporting. For further information on Nature Research policies, see our Editorial Policies and the Editorial Policy Checklist.

Statistics

For all statistical analyses, confirm that the following items are present in the figure legend, table legend, main text, or Methods section.

n/a	Confirmed	
☐	☒	The exact sample size (*n*) for each experimental group/condition, given as a discrete number and unit of measurement
☐	☒	A statement on whether measurements were taken from distinct samples or whether the same sample was measured repeatedly
☐	☒	The statistical test(s) used AND whether they are one- or two-sided<br>*Only common tests should be described solely by name; describe more complex techniques in the Methods section.*
☐	☒	A description of all covariates tested
☐	☒	A description of any assumptions or corrections, such as tests of normality and adjustment for multiple comparisons
☐	☒	A full description of the statistical parameters including central tendency (e.g. means) or other basic estimates (e.g. regression coefficient) AND variation (e.g. standard deviation) or associated estimates of uncertainty (e.g. confidence intervals)
☐	☒	For null hypothesis testing, the test statistic (e.g. *F*, *t*, *r*) with confidence intervals, effect sizes, degrees of freedom and *P* value noted<br>*Give P values as exact values whenever suitable.*
☒	☐	For Bayesian analysis, information on the choice of priors and Markov chain Monte Carlo settings
☐	☒	For hierarchical and complex designs, identification of the appropriate level for tests and full reporting of outcomes
☒	☐	Estimates of effect sizes (e.g. Cohen's *d*, Pearson's *r*), indicating how they were calculated

Our web collection on statistics for biologists contains articles on many of the points above.

Software and code

Policy information about availability of computer code

Data collection	No software was used
Data analysis	FastQC (https://www.bioinformatics.babraham.ac.uk/projects/fastqc/) was used to assess the quality of the raw sequence reads. Reads were then aligned to the Human genome (Hg19) using STAR (version 2.5.2b) (PMID: 23104886) allowing for a maximum of 2 mismatches per 100 bp read. Approximately 85% of reads mapped uniquely to the genome. Read counts per gene were then obtained using HTSeq (PMID: 25260700). Differential gene expression analysis was performed using DESeq2 (PMID: 25516281).

For manuscripts utilizing custom algorithms or software that are central to the research but not yet described in published literature, software must be made available to editors and reviewers. We strongly encourage code deposition in a community repository (e.g. GitHub). See the Nature Research guidelines for submitting code & software for further information.

Data

Policy information about availability of data

All manuscripts must include a data availability statement. This statement should provide the following information, where applicable:

- Accession codes, unique identifiers, or web links for publicly available datasets
- A list of figures that have associated raw data
- A description of any restrictions on data availability

Copied from the Methods section
All relevant data are available in this manuscript and from the corresponding author.
Raw data is deposited in NCBI SRA (BioProject ID: PRJNA830885).

Field-specific reporting

Please select the one below that is the best fit for your research. If you are not sure, read the appropriate sections before making your selection.

☒ Life sciences ☐ Behavioural & social sciences ☐ Ecological, evolutionary & environmental sciences

For a reference copy of the document with all sections, see nature.com/documents/nr-reporting-summary-flat.pdf

Life sciences study design

All studies must disclose on these points even when the disclosure is negative.

Sample size	We determined sample size for our lung cell RNA-sequencing based on power calculations using preexisting similar comparisons of the same analyses on blood-derived MAIT cells.
Data exclusions	No data were excluded from the analyses.
Replication	All attempts at experimental replication, for the data presented in this report, were successful.
Randomization	Samples were allocated into experimental groups randomly.
Blinding	Investigators were not blinded to group allocation in the RNA-seq and flow cytometry analyses. The datapoints collected were not subjective and thus less likely to influences by investigator bias.

Reporting for specific materials, systems and methods

We require information from authors about some types of materials, experimental systems and methods used in many studies. Here, indicate whether each material, system or method listed is relevant to your study. If you are not sure if a list item applies to your research, read the appropriate section before selecting a response.

Materials & experimental systems

n/a	Involved in the study	
☐	☒	Antibodies
☐	☐	Eukaryotic cell lines
☐	☐	Palaeontology and archaeology
☐	☐	Animals and other organisms
☐	☒	Human research participants
☐	☐	Clinical data
☐	☐	Dual use research of concern

Methods

n/a	Involved in the study	
☐	☐	ChIP-seq
☐	☒	Flow cytometry
☐	☐	MRI-based neuroimaging

Antibodies

Antibodies used

Target of Antibody Fluorophore Manufacturer Clone
CD3 PerCP Cy5.5 BioLegend SK7
CD3 PerCP BD Biosciences SK7
CD3 FITC BioLegend UCHT1
CD3 BV421 BioLegend UCHT1
CD4 BV785 BioLegend OKT4
CD8 APC eFluor780 eBioscience SK1
CD8 APC BioLegend HITBa
CD8 FITC BD Biosciences RPA-T8
CD14 APC BioLegend HCD14
TRAV1-2 APC Lewinsohn Lab OF5A12
TCR$^{\alpha\beta}$ PE BD Biosciences T10B9.1A-31
IL-12RB1 PE BD Biosciences 2.4E6
Mouse IgG1 Isotype PE BioLegend MOPC-21
IL-12RB2 APC R&D Systems 305719
Mouse IgG1 Isotype APC BD Biosciences MOPC-21
IFNGR1 FITC R&D Systems 92101
Mouse IgG1 Isotype FITC BioLegend MOPC-21
IL-23R FITC R&D Systems 218213
Mouse IgG2b FITC BioLegend MPC-11
Granzyme A Pac Blue BioLegend CB9
Granzyme B Pac Blue BioLegend QA16A02
Granzyme K APC BioLegend GM26E7

Perforin PE-Cy7 eBioscience (Thermofisher) dG9
Granulysin Alexa Fluor 488 BioLegend DH2
CCR7 PE-Cy7 BioLegend G043H7
Ms IgG2a Isotype PE-Cy7 BioLegend MOPC-173
CD62L PerCP-Cy5.5 BioLegend DREG-56
Ms IgG1 Isotype PerCP-Cy5.5 BioLegend MOPC-21
CD45RO BV421 BioLegend UCHL1
Ms IgG2a Isotype BV421 BioLegend MOPC-173
CD45RA FITC BioLegend HI100
Ms IgG2b Isotype FITC BioLegend MPC-11
Mouse IgG1 Isotype Pac Blue BioLegend MOPC-21
Mouse IgG2b PE-Cy7 BioLegend MPC-11

Validation: Each primary antibody was titrated using mixed negative and positive target cells. Additionally, isotype and FMO controls were used where necessary to determine nonspecific binding by FcR and to set gates in analysis, respectively.

Eukaryotic cell lines

Policy information about cell lines

Cell line source(s): n/a

Authentication: n/a

Mycoplasma contamination: n/a

Commonly misidentified lines (See ICLAC register): n/a

Palaeontology and Archaeology

Specimen provenance: *Provide provenance information for specimens and describe permits that were obtained for the work (including the name of the issuing authority, the date of issue, and any identifying information).*

Specimen deposition: *Indicate where the specimens have been deposited to permit free access by other researchers.*

Dating methods: *If new dates are provided, describe how they were obtained (e.g. collection, storage, sample pretreatment and measurement), where they were obtained (i.e. lab name), the calibration program and the protocol for quality assurance OR state that no new dates are provided.*

☐ Tick this box to confirm that the raw and calibrated dates are available in the paper or in Supplementary Information.

Ethics oversight: *Identify the organization(s) that approved or provided guidance on the study protocol, OR state that no ethical approval or guidance was required and explain why not.*

Note that full information on the approval of the study protocol must also be provided in the manuscript.

Animals and other organisms

Policy information about studies involving animals; ARRIVE guidelines recommended for reporting animal research

Laboratory animals: *For laboratory animals, report species, strain, sex and age OR state that the study did not involve laboratory animals.*

Wild animals: *Provide details on animals observed in or captured in the field; report species, sex and age where possible. Describe how animals were caught and transported and what happened to captive animals after the study (if killed, explain why and describe method; if released, say where and when) OR state that the study did not involve wild animals.*

Field-collected samples: *For laboratory work with field-collected samples, describe all relevant parameters such as housing, maintenance, temperature, photoperiod and end-of-experiment protocol OR state that the study did not involve samples collected from the field.*

Ethics oversight: *Identify the organization(s) that approved or provided guidance on the study protocol, OR state that no ethical approval or guidance was required and explain why not.*

Note that full information on the approval of the study protocol must also be provided in the manuscript.

Human research participants

Policy information about studies involving human research participants

Population characteristics: Supplemental table 1 describes the diverse characteristics of our sample population of tissue donors.

Recruitment	*Describe how participants were recruited. Outline any potential self-selection bias or other biases that may be present and how these are likely to impact results.*
Ethics oversight	All samples were collected and all experiments were conducted under protocols approved by the institutional review board at Oregon Health and Science University. PBMCs were obtained by apheresis from healthy adult donors with informed consent. De-identified lungs not suitable for transplant were obtained from the Pacific Northwest Transplant Bank (PNTB). Our exclusion criteria included significant tobacco smoking history, drowning, crushing chest injuries, lobar pneumonia, and HIV/HBV/HCV infection. Bronchoalveolar lavage fluid for generating T cell clones was obtained under a protocol approved by the University of KwaZulu-Natal Human Biomedical Research Ethics Committee and the Partners Institutional Review Board. The participants provided written informed consent.

Note that full information on the approval of the study protocol must also be provided in the manuscript.

Clinical data

Policy information about clinical studies
All manuscripts should comply with the ICMJE guidelines for publication of clinical research and a completed CONSORT checklist must be included with all submissions.

Clinical trial registration	*Provide the trial registration number from ClinicalTrials.gov or an equivalent agency.*
Study protocol	*Note where the full trial protocol can be accessed OR if not available, explain why.*
Data collection	*Describe the settings and locales of data collection, noting the time periods of recruitment and data collection.*
Outcomes	*Describe how you pre-defined primary and secondary outcome measures and how you assessed these measures.*

Dual use research of concern

Policy information about dual use research of concern

Hazards

Could the accidental, deliberate or reckless misuse of agents or technologies generated in the work, or the application of information presented in the manuscript, pose a threat to:

No	Yes	
☒	☐	Public health
☒	☐	National security
☒	☐	Crops and/or livestock
☒	☐	Ecosystems
☒	☐	Any other significant area

Experiments of concern

Does the work involve any of these experiments of concern:

No	Yes	
☒	☐	Demonstrate how to render a vaccine ineffective
☒	☐	Confer resistance to therapeutically useful antibiotics or antiviral agents
☒	☐	Enhance the virulence of a pathogen or render a nonpathogen virulent
☒	☐	Increase transmissibility of a pathogen
☒	☐	Alter the host range of a pathogen
☒	☐	Enable evasion of diagnostic/detection modalities
☒	☐	Enable the weaponization of a biological agent or toxin
☒	☐	Any other potentially harmful combination of experiments and agents

ChIP-seq

Data deposition

☐ Confirm that both raw and final processed data have been deposited in a public database such as GEO.

☐ Confirm that you have deposited or provided access to graph files (e.g. BED files) for the called peaks.

Data access links<br>*May remain private before publication.*	*For "Initial submission" or "Revised version" documents, provide reviewer access links. For your "Final submission" document, provide a link to the deposited data.*

Files in database submission | *Provide a list of all files available in the database submission.*

Genome browser session (e.g. UCSC) | *Provide a link to an anonymized genome browser session for "Initial submission" and "Revised version" documents only, to enable peer review. Write "no longer applicable" for "Final submission" documents.*

Methodology

Replicates | *Describe the experimental replicates, specifying number, type and replicate agreement.*

Sequencing depth | *Describe the sequencing depth for each experiment, providing the total number of reads, uniquely mapped reads, length of reads and whether they were paired- or single-end.*

Antibodies | *Describe the antibodies used for the ChIP-seq experiments; as applicable, provide supplier name, catalog number, clone name, and lot number.*

Peak calling parameters | *Specify the command line program and parameters used for read mapping and peak calling, including the ChIP, control and index files used.*

Data quality | *Describe the methods used to ensure data quality in full detail, including how many peaks are at FDR 5% and above 5-fold enrichment.*

Software | *Describe the software used to collect and analyze the ChIP-seq data. For custom code that has been deposited into a community repository, provide accession details.*

Flow Cytometry

Plots

Confirm that:

☒ The axis labels state the marker and fluorochrome used (e.g. CD4-FITC).

☒ The axis scales are clearly visible. Include numbers along axes only for bottom left plot of group (a 'group' is an analysis of identical markers).

☒ All plots are contour plots with outliers or pseudocolor plots.

☒ A numerical value for number of cells or percentage (with statistics) is provided.

Methodology

Sample preparation | PBMCs were isolated from the peripheral blood of healthy donors using Ficoll-Paque gradients. Lung single cell suspensions are prepared from recently deceased donor tissue not suitable for transplant from the Pacific Northwest Transplant bank. Small cubes of lung parenchyma, devoid of airway and lymph nodes, were cut into a cold buffer of HBSS (Gibco) media supplemented with HEPES (Gibco) and PSF antibiotic (Sigma). Tissue was then digested for 30 minutes at 37C in a DMEM buffer (Gibco) supplemented with PSF antibiotics (Sigma), elastase (15 µg/mL, Worthington), trypsin I (1.5 µg/mL, Sigma), DNase I (45 µg/mL, Roche). The subsequent suspension was further dissociated using a GentleMACS dissociator (Miltenyi) using the Lung02 program. The single cell suspension is then diluted 1:1 with a buffer of HBSS (Gibco) media supplemented with 2% heat-inactivated fetal bovine serum (Gemini Bio Products), HEPES (Gibco) and PSF antibiotic (Sigma) to dilute homogenate and neutralize digest enzymes. This cell suspension is passed through successive filters in this order: metal mesh sieve filter (size 40 then 60, Sigma), and nylon cell strainer (100 µm then 40 µm, BD Falcon). The resulting cell suspension is washed in RPMI supplemented with 10% heat inactivated pooled human serum and used for experiments or cryo-preserved in heat-inactivated fetal bovine serum with 10% DMSO. Lung-derived T cell clones were isolated from bronchial alveolar lavage (BAL) samples using a protocol described in detail below. Cells to be analyzed for cell surface marker expression were blocked with tetramer staining buffer (PBS buffer containing 2% fetal bovine serum). PBMCs were stained with the MR1/5-OP-RU tetramer (NIH Tetramer core facility) at 0.3 nM in 25 µL volume for 45 minutes in tetramer staining buffer at room temperature in the dark. Viability and surface stains were added on top of the tetramer stain for another 20 min at 4°C in the dark. Samples were then washed in tetramer staining buffer. For intracellular staining, cells were permeabilized and fixed using the CytoFix/CytoPerm kit (BD) as directed. Antibodies to intracellular proteins were added to samples for 20 minutes at 4°C in the dark and then washed with PermWash (BD).

Instrument | LSRFortessa flow cytometer (BD) or a CytoFLEX S flow cytometer (Beckman Coulter)

Software | FlowJo

Cell population abundance | Each FACS sample was verified to be over 98% pure for the targeted cell type.

Gating strategy | Live, CD45+, CD4 negative, CD14/CD20/EpCAM negative were sorted into four populations: MR1/5-OP-RU+ CD8+, MR1/5-OP-RU+ CD8 negative, MR1/5-OP-RU-negative CD8+, and MR1/5-OP-RU-negative CD8 negative.

☒ Tick this box to confirm that a figure exemplifying the gating strategy is provided in the Supplementary Information.

Magnetic resonance imaging

Experimental design

Design type	*Indicate task or resting state; event-related or block design.*
Design specifications	*Specify the number of blocks, trials or experimental units per session and/or subject, and specify the length of each trial or block (if trials are blocked) and interval between trials.*
Behavioral performance measures	*State number and/or type of variables recorded (e.g. correct button press, response time) and what statistics were used to establish that the subjects were performing the task as expected (e.g. mean, range, and/or standard deviation across subjects).*

Acquisition

Imaging type(s)	*Specify: functional, structural, diffusion, perfusion.*
Field strength	*Specify in Tesla*
Sequence & imaging parameters	*Specify the pulse sequence type (gradient echo, spin echo, etc.), imaging type (EPI, spiral, etc.), field of view, matrix size, slice thickness, orientation and TE/TR/flip angle.*
Area of acquisition	*State whether a whole brain scan was used OR define the area of acquisition, describing how the region was determined.*

Diffusion MRI ☐ Used ☐ Not used

Preprocessing

Preprocessing software	*Provide detail on software version and revision number and on specific parameters (model/functions, brain extraction, segmentation, smoothing kernel size, etc.).*
Normalization	*If data were normalized/standardized, describe the approach(es): specify linear or non-linear and define image types used for transformation OR indicate that data were not normalized and explain rationale for lack of normalization.*
Normalization template	*Describe the template used for normalization/transformation, specifying subject space or group standardized space (e.g. original Talairach, MNI305, ICBM152) OR indicate that the data were not normalized.*
Noise and artifact removal	*Describe your procedure(s) for artifact and structured noise removal, specifying motion parameters, tissue signals and physiological signals (heart rate, respiration).*
Volume censoring	*Define your software and/or method and criteria for volume censoring, and state the extent of such censoring.*

Statistical modeling & inference

Model type and settings	*Specify type (mass univariate, multivariate, RSA, predictive, etc.) and describe essential details of the model at the first and second levels (e.g. fixed, random or mixed effects; drift or auto-correlation).*
Effect(s) tested	*Define precise effect in terms of the task or stimulus conditions instead of psychological concepts and indicate whether ANOVA or factorial designs were used.*

Specify type of analysis: ☐ Whole brain ☐ ROI-based ☐ Both

Statistic type for inference (See Eklund et al. 2016)	*Specify voxel-wise or cluster-wise and report all relevant parameters for cluster-wise methods.*
Correction	*Describe the type of correction and how it is obtained for multiple comparisons (e.g. FWE, FDR, permutation or Monte Carlo).*

Models & analysis

n/a	Involved in the study
☐	☐ Functional and/or effective connectivity
☐	☐ Graph analysis
☐	☐ Multivariate modeling or predictive analysis

Functional and/or effective connectivity	*Report the measures of dependence used and the model details (e.g. Pearson correlation, partial correlation, mutual information).*
Graph analysis	*Report the dependent variable and connectivity measure, specifying weighted graph or binarized graph, subject- or group-level, and the global and/or node summaries used (e.g. clustering coefficient, efficiency, etc.).*

Multivariate modeling and predictive analysis	*Specify independent variables, features extraction and dimension reduction, model, training and evaluation metrics.*
